# Predicting immunotherapy benefit in leiomyosarcoma through active chromatin cfDNA profiling

**DOI:** 10.1038/s41698-026-01451-9

**Published:** 2026-05-05

**Authors:** Carlos Diego Holanda Lopes, Hsin-Ta Wu, Katharine Dilger, Yue Wendy Zhang, Abdulazeez Salawu, Lee-Anne Stayner, Abha A. Gupta, Aaron R. Hansen, Anna Spreafico, Philippe L. Bedard, Maggie C. Louie, Lillian L. Siu, Albiruni R. Abdul Razak

**Affiliations:** 1 Princess Margaret Cancer Centre, University Health Network, Toronto, ON Canada; 2 Sinai Health System, University Health Network, Toronto, ON Canada; 3 Aqtual, Hayward, CA USA; 4 Princess Margaret Cancer Consortium, Marathon of Hope Cancer Centres Network, Toronto, ON Canada

**Keywords:** Biomarkers, Cancer, Computational biology and bioinformatics, Oncology

## Abstract

Leiomyosarcoma (LMS) is an aggressive soft-tissue sarcoma characterized by epigenomic dysregulation and variable responsiveness to checkpoint inhibitors (CPIs). In this study, we evaluated a novel liquid biopsy platform based on circulating cell-free DNA active chromatin (cfDNA_ac_) profiling to identify baseline biomarkers associated with clinical benefit rate (CBR) in 30 LMS patients treated with durvalumab plus olaparib or cediranib. A total of 1,570 molecular features across genomic regions were identified, and recursive feature elimination was applied to select discriminative cfDNA_ac_ signatures. Patients achieving CBR demonstrated enrichment of pathways related to cell death, interferon-γ signaling, and immune activation. Signatures reflecting B-cell activation, T-cell activation, and extracellular matrix organization were significantly associated with improved progression-free survival (PFS; p < 0.05). In contrast, a baseline tumor fraction >5% was negatively associated with CBR (p = 0.041) and correlated with inferior PFS (p = 0.008). Distinct copy number variation profiles further characterized patients with CBR and were significantly associated with PFS. In this analysis, cfDNA_ac_ profiling represents a promising non-invasive strategy to predict clinical benefit from CPI-based therapy in LMS. Prospective validation in independent cohorts is warranted to clarify its clinical utility.

## Introduction

Leiomyosarcoma (LMS) is a rare mesenchymal neoplasm that most commonly occurs in soft tissues and, less frequently, in bone. Despite its rarity, it accounts for approximately 20% of all new cases of soft tissue sarcomas (STS)^1^. In the metastatic setting, the prognosis is generally poor with only 7–10% of patients surviving at 5 years^2^. Systemic treatment options are limited to single-agent and combination of cytotoxic chemotherapy or targeted therapies, which are associated with modest benefits^2^. The use of checkpoint inhibitors (CPI) holds promise in some STS such as undifferentiated pleomorphic sarcomas, alveolar soft part sarcomas, and cutaneous angiosarcomas^3,4^. Furthermore, combinatorial approaches involving CPI or targeted therapies, particularly in the context of LMS, have demonstrated promising clinical activity^5,6^. The presence of tertiary lymphoid structures^7^, the profile of tumor-infiltrating T lymphocytes^8^, and tumor immunogenic clusters based on transcriptomic analysis^9^, have been identified as potentially predictive biomarkers of clinical benefit in the context of immunotherapy. LMS, as in some other STS, are characterized by a low frequency of actionable mutations and a low mutational burden. In contrast, alterations in epigenetic pathway, particularly in those related to chromatin remodeling and histone modification, are observed in over 50% of LMS^10^. Despite these genomic insights, a notable gap remains in understanding how these epigenetic features can correlate with the efficacy of CPI treatments in LMS and in other STS.

The DAPPER trial (phase 2, single-center, randomized, multi-cohort; NCT03851614) enrolled patients with advanced mismatch repair–proficient colorectal cancer, pancreatic adenocarcinoma, and LMS who had progressed after first-line treatment. Participants were randomized (1:1) to durvalumab combined with either olaparib (a poly [ADP-ribose] polymerase [PARP] inhibitor) or cediranib (an antiangiogenic small-molecule agent) until progression or limited toxicity^11,12^. In the LMS cohort (*n* = 30), some patients achieved disease stabilization or shrinkage across both arms^13^. High levels of type 1 macrophage and B-cell transcriptomic signatures correlated with improved overall survival (OS), thus representing as potential biomarkers^13^. The trial also collected tumor and blood samples longitudinally to enable additional correlative analyses.

Given the prevalence of epigenetic alterations in LMS, we conducted a correlative study using a novel liquid biopsy platform to isolate cell-free DNA associated with active chromatin (cfDNA_ac_). Unlike conventional cfDNA, cfDNA_ac_ is bound to regulatory protein complexes^14^ that mark transcriptionally active chromatin regions, providing a unique view of the tumor’s regulatory and transcriptional landscape.

In this study, we hypothesized that cfDNA_ac_ profiling may enable the identification of molecular features associated with clinical benefit from checkpoint inhibitor–based therapy in patients with metastatic LMS enrolled in the DAPPER trial (Supplementary Fig. 1).

## Results

### Summary of the DAPPER Trial – LMS cohort

Baseline clinical characteristics of the 30 patients in the LMS cohort are summarized in Supplementary Data 1. Most were female (93.3%, *n* = 28) with a median age of 54.5 years (range: 39–72). Tumor origin was uterine in 60% (*n* = 18) and extra-uterine in 40% (*n* = 12). Across both treatment arms, patients received a median of 3 cycles of CPI-based therapy (range: 1–13). After a median follow-up of 15.2 months (range: 1.8–62.7), median PFS was 2.8 months (95% CI: 2.8–5.4), and median OS was 15.3 months (95% CI: 14.7–15.4). CBR was observed in 26.7% (*n* = 8), with 40.0% (*n* = 6) in Arm A and 13.3% (*n* = 2) in Arm B.

### cfDNA_ac_ Analysis and Stratification of Clinical Benefit

cfDNA_ac_ was successfully extracted from baseline plasma of all LMS patients (*n* = 30) using a novel chromatin-capture method^14^. Following library preparation, sequencing reads were deconvoluted into cfDNA_ac_ and nucleosomal (cfDNA_nuc_) fractions. Signal enrichment across regulatory regions, including exons, promoters, enhancers, and TFBS, was analyzed to identify features associated with clinical benefit.

**Fig. 1:**
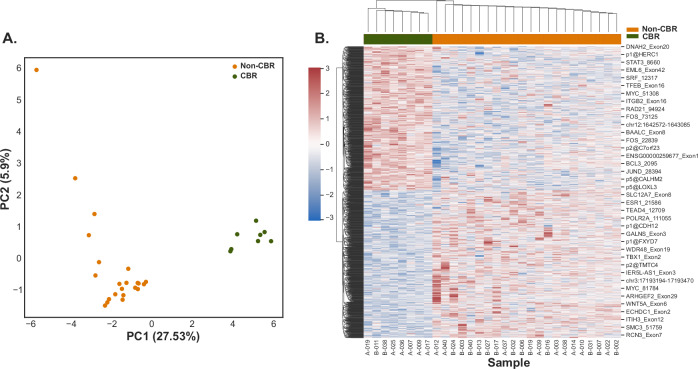
cfDNA_ac_ feature selection distinguishes patients with and without CBR. **A** Principal component analysis was performed using 1,570 selected molecular features between the CBR (*n* = 8) and non-CBR (*n* = 22) groups. The first two principal components (PC1 and PC2), which explain 33.4% of the total variance and are shown in the plot. Each point represents an individual sample, colored according to clinical response group: CBR (green), non-CBR (orange). **B** Hierarchical clustering of the heatmap based on selected cfDNA_ac_ signals shows concordance with the CBR and non-CBR groups. Legend: CBR – clinical benefit rate.

Univariate analysis followed by recursive feature elimination identified 1,570 cfDNA_ac_ features linked to CBR (*p* < 0.01; Supplementary Fig. 2A). Notably, the volcano plot revealed significant differential enrichment of molecular features in patients who achieved CBR (*n* = 8) compared with those who did not (*n* = 22) (Supplementary Fig. 2B). Principal component analysis of the selected features demonstrated separation between CBR and non-CBR groups, with the first two components accounting for 33.4% of total variance (Fig. 1A). Consistently, hierarchical clustering based on these cfDNA_ac_ features showed strong concordance with clinical benefit categories (Fig. 1B).

**Fig. 2:**
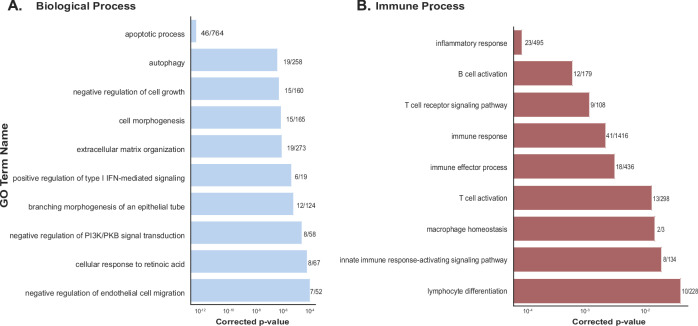
Pathway enrichment analysis of cfDNA_ac_-derived features in patients with CBR. Over-representation analysis of Gene Ontology **A** biological processes and **B** immune-related gene sets reveal significant enrichment of cell death pathways, interferon-gamma signaling, and activation of innate and adaptive immune responses in CBR patients. Legend: CBR – clinical benefit rate.

Having established that cfDNA_ac_ profiling could discriminate between patients with and without clinical benefit, we next explored the biological and immunological processes underlying these differences through pathway enrichment analysis using GO and immune-related gene set databases (Fig. 2). Within the GO biological process category, 1,055 gene sets were significantly enriched in the CBR group compared to the non-CBR group. Enrichment was assessed using Fisher’s exact test, and significance was defined as a p-value adjusted for FDR < 0.05. Notably, these pathways involved negative regulation of cell proliferation, cell death, and type I interferon signaling (p_adj_ < 0.05; Fig. 2A). Moreover, analysis of immune-related biological processes identified 18 pathways significantly enriched in the CBR group, predominantly reflecting activation of both innate and adaptive immune responses (p_adj_ < 0.05; Fig. 2B).

### Specific cfDNA_ac_-Derived Immune-Enriched Signatures Correlate with PFS

**Fig. 3:**
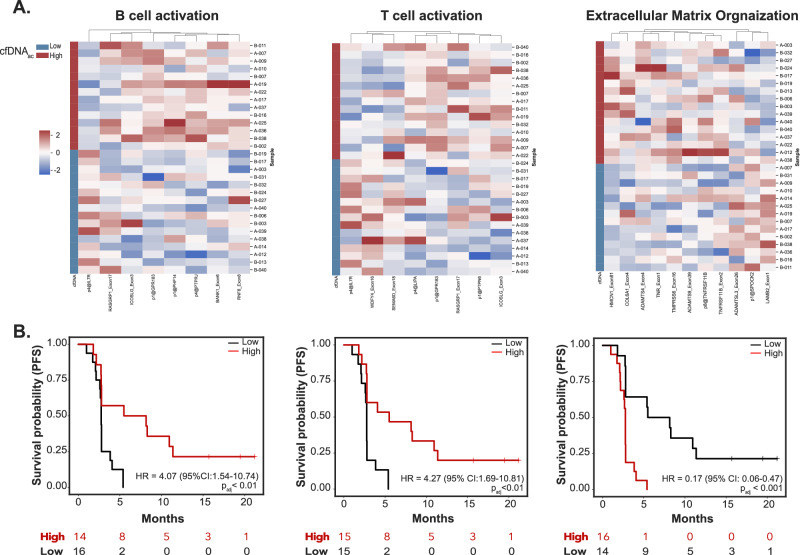
cfDNA_ac_-derived immune signatures are associated with PFS. Immune-enriched signatures based on cfDNA_ac_ signals and clinical outcomes. **A** Heatmaps show the distribution of patient clinical outcomes stratified by B cell activation, T cell activation, and extracellular matrix organization signatures (superior part, from the left to the right respectively). All signatures are significantly associated with PFS after adjusting for FDR using the Benjamini–Hochberg method (p < 0.05). **B** Corresponding Kaplan–Meier curves illustrating PFS associations are shown below each heatmap. Legend: PFS – progression-free survival; FDR - false discovery rate.

As previously reported, B-cell and T-cell activation signatures have been proposed as biomarkers of response to CPI therapy^7,8^. Building on this, we examined whether cfDNA_ac_-derived profiles are enriched for immune-related signatures associated with PFS. Using principal component analysis (PCA; *n* = 2) on cfDNA_ac_ signal intensities across immune-related gene sets, samples were stratified into two groups. These groups were classified as high or low cfDNA_ac_ based on the mean values of the k-means centroids and assessed by the Kaplan Meier method. Interestingly, an enrichment of B-cell activation (HR: 4.07; 95% CI: 1.54–10.74; *p* < 0.01) and T-cell activation (HR: 4.27; 95% CI: 1.69–10.81; *p* < 0.01) signatures were significantly associated with a higher likelihood of prolonged PFS exceeding 12 months (Fig. 3A, B). In contrast, enrichment of extracellular matrix organization signature was linked with a reduced likelihood of this PFS outcome (HR: 0.17; 95% CI: 0.06–0.47; *p* < 0.001; Fig. 3A, B). Additional cfDNA_ac_-derived gene set signatures, including high expression of lymphocyte differentiation and apoptotic process signatures, as well as reduction regulation of cell growth–related signals, were also significantly associated with a higher likelihood of prolonged PFS (Supplementary Fig. 3A and 3B). Other signatures with statistically significant associations with PFS are presented in Supplementary Data 2.

### cfDNA_ac_-Derived CNV Profiles and Clinical Outcomes

**Fig. 4:**
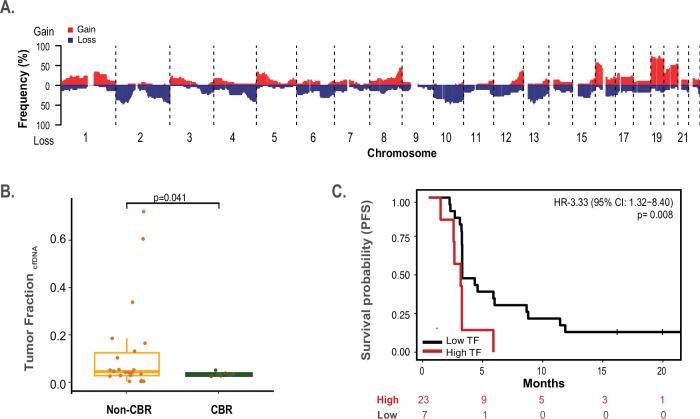
Baseline tumor fraction and CNV profiles are associated with clinical outcomes. **A** CNV profiles derived from cfDNA_ac_ (Top) closely reflect those from tumor tissue gDNA. Higher tumor fraction in baseline plasma cfDNA_ac_ is associated with reduced CBR **B** and shorter PFS **C**. Tumor fraction groups were defined using a 5% threshold as high vs low. Legend: CBR – clinical benefit rate; CNV – copy number variation; PFS – progression-free survival. CBR and non-CBR groups had 8 and 22 patients, respectively.

Given that the cfDNA_ac_ assay is based on whole genome sequencing, we assessed CNVs derived from cfDNA to investigate their associations with CBR and PFS. Consistent with recurrent CNV patterns previously reported from tumor tissue genomic DNA^15^, cfDNAac-derived CNV profiles exhibited similar hallmark amplification and deletion events characteristic of LMS, supporting the ability of the assay to capture tumor-derived CNV features from plasma (Fig. 4A). Patients with higher baseline tumor fractions ( > 5%) were significantly less likely to achieve clinical benefit (p = 0.041 two-sided Welch’s t-test; Fig. 4B) and experienced shorter PFS (HR: 3.33; 95% CI: 1.32–8.40; p = 0.008; Fig. 4C), highlighting tumor burden as a negative prognostic factor. Since very high–tumor-fraction samples are dominated by large clonal CNVs, which can obscure subtle response-associated patterns, these samples were excluded from the initial discovery of CNV regions to avoid being confounded by tumor fraction.

To achieve higher-resolution analysis, CNVs were assessed in 1 Mb genomic segments (bins) across the genome, with gains and losses evaluated separately. Odds ratios (OR) for CNV events between CBR (*n* = 8) and non-CBR (*n* = 22) groups were calculated for each segment. Contiguous segments with OR ≥ 5 or ≤ 0.2, observed in at least three patients harboring CNVs, were merged to define regions of interest with potential prognostic value. These thresholds, reflecting strong effect sizes ( ≥ 5-fold enrichment or depletion), identified loci that differed between CBR and non-CBR groups while minimizing noise from marginal events.

**Fig. 5:**
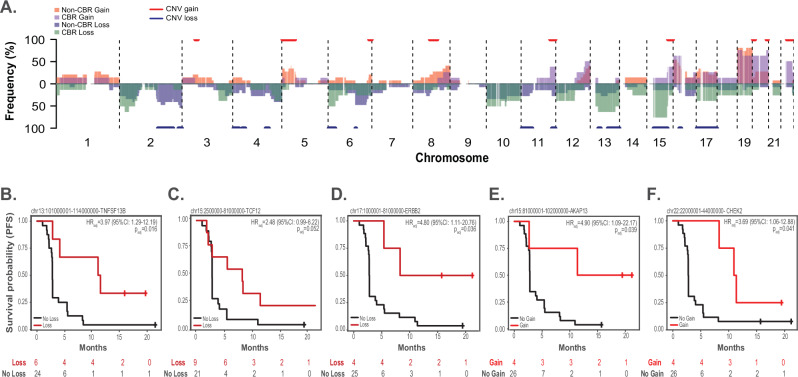
Distinct CNV profiles were derived for non-CBR and CBR samples. **A** Genomic segmentation highlights regions with differential copy number gains (top) and losses (bottom) between groups. **B–F** Segments significantly associated with clinical benefit were further analyzed using Cox regression to evaluate their association with PFS, with hazard ratios and p-values adjusted for tumor fraction to account for baseline tumor burden differences. Representative cancer-related genes within identified segments are noted on KM plots. Legend: CNV – copy number variation; PFS – progression-free survival.

We next evaluated whether these CBR-associated CNV regions were also prognostic for longer-term outcomes. CNV profiles were analyzed for associations with 12-month PFS using Cox proportional hazards regression models adjusted for tumor fraction and applied across the full cohort (Fig. 5A). This ensured that CNV regions identified using the tumor-fraction filtered discovery set were subsequently evaluated for prognostic relevance in all available baseline samples.

Although CBR (assessed at 6 months) and PFS (assessed at 12 months) are related, CBR provides a short-term binary classification, whereas PFS captures durable clinical benefit. Evaluating CNVs against PFS, therefore, allowed us to determine whether loci distinguishing CBR status also carried independent prognostic value. Several CNV-defined regions discovered in the CBR analysis were significantly associated with prolonged PFS, including deletions on chromosomes 13, 15, and 17 (Fig. 5B–D) and amplifications on chromosomes 15 and 22 (Fig. 5E–F). Furthermore, multiple cancer-associated genes located within these CNV regions, such as *TNFSF13B, TCF12, ERBB2, AKAP13*, and *CHEK2*, also demonstrated significant associations with PFS (Fig. 5B–F; Supplementary Data 3 and Supplementary Data 4), underscoring the biological relevance of these loci.

## Discussion

Despite many clinical trials, doxorubicin monotherapy remains the global standard first-line treatment for advanced LMS and most STS^2,16,17^. More recently, the combination of doxorubicin and trabectedin demonstrated an OS benefit as first-line therapy for advanced LMS. However, its clinical utility is limited by a high incidence of grade 3–4 toxicities^18^. Several single agents and combinations have been evaluated in subsequent lines of treatment for STS, but they have provided limited clinical benefit^19–21^. The use of CPI in LMS and STS is still limited, underscoring the importance of correlative analyses from CPI trials to support the development of predictive biomarkers for improved patient selection.

This post hoc analysis is the first study to demonstrate that non-invasive profiling of baseline genomic features in LMS using a novel active chromatin cfDNA (cfDNA_ac_) technology is feasible and may help identify patients more likely to benefit from CPI therapy. LMS is a biologically heterogeneous disease, and prior efforts to define molecular subtypes with prognostic relevance have leveraged multi-omic approaches^22–26^. However, these strategies typically depend on tumor biopsies or surgical specimens, which can carry procedural risks.

Emerging evidence suggests that various sarcoma subtypes shed tumor-derived genetic material into the bloodstream, supporting the use of liquid biopsy–based assays for applications including diagnosis^27^, minimal residual disease detection^28,29^, response monitoring^30^, and individualized therapeutic targeting^31^. However, sarcomas are a heterogeneous group of malignancies characterized by diverse genomic alterations (most notably CNVs and translocations)^32^ which limit the sensitivity of conventional tumor-informed approaches in detecting alterations absent from the primary tumor or acquired during treatment^33^. These limitations are addressed by our tumor-agnostic liquid biopsy approach, which leverages whole-genome sequencing to successfully profile cfDNA_ac_ in all samples.

cfDNA fragments originating from active chromatin carry noncanonical fragmentation patterns distinct from the classical apoptotic nucleosomal ladder. These fragments, often described as longer irregular cfDNA fragments, are observed across diverse physiological and pathological states^34–36^. Additionally, fragmentation patterns arise from multiple processes, including chromatin accessibility, transcription factor occupancy, nuclease activity, and clearance kinetics, rather than from apoptosis alone, and reflect disease-specific influences that shape the fragment landscape in plasma^37–39^. As a result, assays that rely on longer cfDNA fragments may expand detection of signals from active regulatory regions and capture a broader spectrum of biological activity. Accordingly, approaches that directly enrich for cfDNA_ac_ provide a clear advantage for detecting disease-specific signals, as dysregulated genes are more frequently located within open, transcriptionally active chromatin than within tightly packed nucleosomal regions^14,39,40^.

Traditional chromatin-mapping methods, such as ChIP-seq^41^ and ATAC-seq^42^, require an intact nuclei and are therefore incompatible with current cell-free DNA analysis. More recently, cell-free ChIP-seq has shown that immunoprecipitation of histone marks such as H3K4me3 and H3K27ac from plasma can recover biologically meaningful regulatory information^43,44^, although it is limited by antibody dependence, which reduces scalability and the ability to discover novel chromatin states. In light of these limitations, our cfDNA_ac_ approach overcomes these challenges by capturing active chromatin fragments without prior knowledge of specific epigenetic marks, thereby enabling more comprehensive profiling of gene regulation and expression. Notably, promoter-level cfDNA_ac_ signals correlate strongly with tumor RNA-seq data in both our prior study^45^ and the current sarcoma cohort (rp = 0.81; Supplementary Fig. 4), demonstrating the ability of cfDNA_ac_ to capture tumor gene expression. This is particularly valuable in advanced-stage cancers, where tumor tissue is often inaccessible, limiting the feasibility of RNA-based profiling. The ability to noninvasively and dynamically capture epigenetic and transcriptional activity in plasma may offer new opportunities for disease diagnosis, prognosis, and monitoring of treatment response.

Recognizing that CPI efficacy in STS does not follow a one-size-fits-all paradigm, recent trials have focused on histological subtypes with evidence of immunotherapy responsiveness. In a phase 1b trial, patients with metastatic LMS, angiosarcoma, undifferentiated pleomorphic sarcoma, and others received botensilimab (anti–CTLA-4) with or without balstilimab (anti–PD-1), resulting in a 19.2% ORR and median response duration of 21.7 months in the overall STS population^46^. Responses were also seen in LMS patients^46^. Another phase Ib trial in metastatic LMS reported a 56.6% ORR with doxorubicin, trabectedin, and nivolumab, in which increased circulating levels of high mobility group box 1 (*HMGB1*), a nuclear DNA-binding protein released during immunogenic cell death, correlated with improved PFS^47^. While histology-driven trial enrollment improves response rates, biomarker-based stratification may further refine patient selection.

Analysis of cfDNA_ac_ signals revealed enrichment of biologically relevant pathways in patients who achieved clinical benefit, particularly those linked to immune activation and tumor cell death. Consistent with the original DAPPER study^12^ and other independent reports^7,8^, patients who derived clinical benefit showed increased B-cell and T-cell activation signatures, whereas extracellular matrix organization displayed an inverse association. This observation also aligns with prior evidence indicating that enrichment of extracellular matrix organization signature correlates with resistance to checkpoint inhibitor therapy, likely reflecting a tumor microenvironment that is more permissive of immune exclusion and protective for tumor cells^48^. These findings support the biological validity of cfDNA_ac_-based profiling and its potential to capture key tumor and immune features predictive of response.

In addition to transcriptomic insights, this cfDNA_ac_ platform generated genome-wide CNV profiles that closely mirrored previously reported tumor tissue data^23^. Leveraging these CNV profiles, we estimated the circulating tumor fraction and identified a 5% threshold that effectively stratified patients by clinical benefit and PFS. This lower threshold is notable in the context of prior studies, such as those using the FoundationOne Liquid CDx assay, in which a 10% tumor fraction cutoff has been shown to be an independent prognostic factor across multiple tumor types, likely reflecting more aggressive disease biology^49^. Similarly, in metastatic STS, a tumor fraction ≥10% has been associated with significantly increased mortality risk^50^. Our results reinforce the prognostic value of tumor fraction in CPI-treated LMS, with a lower predictive threshold of 5%, potentially reflecting enhanced assay sensitivity or distinct biological characteristics of LMS.

We also identified distinct CNV profiles between patients with and without clinical benefit, with the non-CBR group exhibiting a higher overall CNV burden. These differences were further supported by differential gains or losses at specific chromosomal regions associated with PFS. Several genes within these CNV regions are linked to tumor immunity. For example, *TNFSF13B (BAFF)* regulates B-cell survival and supports antigen presentation and T-cell priming^51,52^, so its loss could reflect reduced local humoral immune support. Similarly, *IL2RB (CD122)* is essential for IL-2–driven expansion and persistence of cytotoxic lymphocytes, suggesting that copy number gain might influence T-cell fitness or trafficking^53^. There is also emerging evidence that loss of *TCF12* in tumor cells may shift the immune microenvironment toward a less immunosuppressive state by lowering expression of inhibitory mediators and reducing recruitment of suppressive myeloid cells^54^. In contrast to these immune-regulatory effects, *CHEK2*, *ERBB2*, and *AKAP1* are primarily involved in DNA damage response, proliferation, or replication, and likely influence tumor immunity indirectly through changes in tumor survival and proliferation.

Consistent with previous reports, higher CNV burden has been associated with reduced immune pathway activity and inferior outcomes following CPI therapy^55,56^. Specifically, in a cohort of non–small cell lung cancer patients receiving immunotherapy with or without chemotherapy, those with low plasma CNV burden (defined as <0.10) demonstrated improved OS, PFS, and response rates^55^. Similarly, in hepatobiliary cancers treated with CPIs, low cfDNA-derived CNV scores were associated with better OS and PFS, whereas this relationship was not evident with non-immunotherapy regimens^56^. In another study, integrated genomic analysis of intimal sarcomas, a rare vascular-origin subtype of STS, identified two molecular subtypes, CNV-high (CNV-H) and MSI-H–like. The CNV-H subtype was characterized by reduced immune gene expression, lower enrichment for immune-related pathways, and immune-desert histology, which correlated with inferior OS (47.4 vs. 83.8 months)^57^. Together, these findings highlight the emerging role of CNV profiles as potential predictive biomarkers in CPI-treated LMS and STS.

This study has several limitations, including a small number of patients, potential selection bias inherent in a single-center cohort, the exploratory nature of a post hoc analysis, and the absence of an independent external validation cohort to confirm these results. In light of these limitations, efforts are underway to extend these analyses to independent LMS cohorts. Moreover, assessing the feasibility of the cfDNA_ac_ approach for transcriptomic-based characterization of LMS molecular subtypes, as previously defined using tumor tissue^22–26^, and determining how cfDNA_ac_-derived biomarkers are distributed across these subtypes represent important directions for future investigation. These factors, along with the non-comparative study design, also constrained our ability to assess overall survival. Although women constituted the majority of the cohort, we do not believe that this sex imbalance materially influenced cfDNA_ac_ shedding and signal detection. The cfDNA_ac_ platform has previously demonstrated robust performance in distinguishing healthy individuals from patients with colorectal cancer in a study that included a balanced representation of male and female participants, supporting the sex-independent technical reliability of this approach^45^. Consistent with prior reports on later-line LMS treatments^19–21^, only a small number of patients achieved partial responses, making it challenging to clearly distinguish between groups with and without clinical benefit. In addition, the predominance of uterine-origin LMS in our cohort, which has previously been described as having a colder immune environment than the other non-uterine subtypes^58^ and exhibiting lower sensitivity to systemic checkpoint inhibitors^59,60^, may have further contributed to the modest objective response rates observed.

Nevertheless, our analysis successfully differentiated these two patient populations despite these limitations. Importantly, we identified enrichment of immune activation pathways in patients who achieved CBR, supporting the hypothesis that these signals are more likely attributable to ongoing CPI-based treatment activity.

Our results provide a rationale for incorporating cfDNA_ac_ profiling into future prospective and adaptive clinical trial designs in leiomyosarcoma. cfDNA_ac_-derived immune and genomic signatures could help stratify patients for immunotherapy-based combination regimens, whereas patients lacking these features could be directed to standard-of-care therapies, with the aim of improving patient selection and optimizing therapeutic benefit in LMS and potentially other sarcoma subtypes.

Our findings demonstrate that capture and analysis of cfDNA_ac_ is a feasible, non-invasive approach for identifying patients with metastatic LMS who are more likely to benefit from CPI-based therapies. The enrichment of immune-related and cell death–associated pathways underscores the biological relevance of this platform. Moreover, cfDNA_ac_-derived biomarkers, including T-cell and B-cell activation, and extracellular matrix remodeling signatures, CNV profiles, and circulating tumor fraction, may enhance patient stratification prior to treatment initiation. These results warrant further evaluation across other sarcoma subtypes treated with immunotherapy to assess their broader applicability within STS. Prospective validation is needed to establish cfDNA_ac_ as a precision medicine tool that can be integrated into clinical practice.

## Methods

### DAPPER Study Design – LMS cohort

This is a post hoc analysis of the LMS cohort from the DAPPER trial, for which the primary results have been previously reported^13^. Eligible patients were adults ( ≥ 18 years) with histologically confirmed metastatic or unresectable LMS, measurable disease per RECIST v1.1, ECOG performance status ≤1, and disease progression following at least one prior systemic therapy. Patients were randomized to receive durvalumab (1,500 mg every 4 weeks by cycle) with either olaparib (300 mg twice a day; Arm A) or cediranib (20 mg daily, 5 days on/2 days off; Arm B). Treatment continued until progression, unacceptable toxicity, 12 months, or consent withdrawal. Co-primary endpoints were multiplex IHC analysis of CD4 + /CD8 + T cells and treatment-related adverse events. Secondary endpoints included objective response rate (ORR), clinical benefit rate (CBR; patients achieving partial response or stable disease ≥6 months), progression-free survival (PFS) (according to RECIST v1.1^61^ and iRECIST^62^), and OS.

### Active Chromatin Detection and Extraction

Plasma samples (*n* = 30) were collected at baseline and paired tumor biopsies for RNA-seq were obtained when feasible. Plasma samples (2 mL) were processed and used to extract and enrich cfDNA_ac_, followed by library preparation as previously described^14^. Samples that passed quality control (QC) were pooled and sequenced on the NovaSeq 6000 platform (Illumina).

### Genomic Feature Selection

Sequencing reads were aligned to the human reference genome (hg19), and fragments were deconvoluted into cfDNA_ac_ and nucleosomal cfDNA (cfDNA_nuc_) as previously described^14^. Fragment counts were quantified at predefined genomic features: promoters, exons, enhancers, and transcription factor binding sites (TFBS). Features were excluded if they overlapped genomic regions with low mappability, as defined by a score < 1 in the ENCODE 75-mer mappability track^63,64^, or if the number of reads in the region was fewer than 20. Feature lengths were normalized using a Robust Linear Model (RLM)^65^ when lengths varied across features, and GC bias was corrected using LOWESS smoothing. Finally, quantile normalization was applied to standardize feature distributions across samples.

Feature selection was performed using the Mann-Whitney U test (p < 0.01), followed by recursive feature elimination (RFE) to identify cfDNA_ac_ features that distinguish patients with CBR from those without (non-CBR). A total of 1,570 features were selected: promoters (*n* = 305), exons (*n* = 613), enhancers (*n* = 95), and TFBS (*n* = 557), as shown in Supplementary Data 5. Promoter features follow the format *pX@transcript_ID*, where *pX* denotes the indexed promoter region associated with a transcript and *transcript_ID* corresponds to the UCSC Genome Browser transcript accession used for genomic annotation^66^.

### Pathway Enrichment Analysis

Over-representation analysis (ORA) was performed using Gene Ontology (GO) biological process terms and curated immune-related gene sets^67,68^. Enrichment was assessed using Fisher’s exact test, with p-values adjusted using the Benjamini–Hochberg method.

To assess the prognostic relevance of cfDNA_ac_ signals within enriched immune gene sets, principal component analysis (PCA) was performed on each gene set, followed by multiple-start (*n* = 10) k-means clustering on the first two principal components. Samples were classified as “high” or “low” signal based on cluster centroid means. Adjusted Rand Index (ARI) across 50 random seeds, demonstrating high reproducibility across runs, with a mean ARI of 0.97 across gene sets. PFS was assessed as a dichotomous variable at 12 months based on RECIST v1.1 criteria: patients without progression or death were assigned a status of 0, and those with progression or death, a status of 1. OS was extracted from clinical records (0 = alive, 1 = deceased).

### Copy Number Variation Analysis

Genome-wide read coverage profiles in Wiggle (WIG) format (a text-based file format developed by the University of California, Santa Cruz [UCSC] Genome Browser for representing continuous data across genomic coordinates) were generated from aligned fragments using fixed length 1 megabase (Mb) segment across all chromosomes. Copy number variation (CNV) analysis was performed with ichorCNA (v0.3.2,)^69^ using a panel of in-house panel of healthy controls (Aqtual, Inc.) to model background noise and enhance CNV call specificity.

For the initial CNV discovery analysis, samples with exceptionally high tumor fraction ( ≥ 5%) were excluded. Although a high tumor fraction can increase sensitivity for detecting tumor-derived alterations, our objective at this stage was to identify CNV regions that are differentially associated with clinical benefit response (CBR vs. non-CBR) without confounding from tumor purity. In high–tumor-fraction samples, large clonal CNVs dominate the cfDNA signal, producing exaggerated amplitudes and altered variance structures that can bias normalization and segmentation and mask subtle, response-associated CNV patterns. Excluding these samples during region discovery ensured that identified CNV features reflected biologically meaningful differences between CBR and non-CBR groups rather than differences in tumor burden.

CNV frequencies and odds ratios for amplifications and deletions were calculated for each 1 Mb bin using 2×2 contingency tables stratified by CBR status. Differential CNV segments were defined as consecutive bins with odds ratios ≥5 or ≤0.2, corresponding to approximately five-fold differences in odds between groups. These thresholds were used as conservative effect-size cutoffs to prioritize bins showing strong enrichment or depletion across contiguous genomic regions. After response-associated CNV regions were defined, all samples, including those with high tumor fraction, were included in survival analyses. Associations between CNV segments and PFS were assessed using Cox proportional hazards models (R Survival package v3.5.8), stratified by CNV status and adjusted for tumor fraction across all 30 baseline samples. Representative genes within clinically relevant segments were identified and evaluated for PFS associations using the same modeling approach.

All analyses were performed using R software (version 4.3.1). Statistical significance was defined as two-sided *p* < 0.05.

### Human Ethics and Consent to Participate Declarations

The DAPPER trial, including tissue and blood sample collection for correlative analysis, received approval (Reference number: 18-6093) from the Research Ethics Board at the Princess Margaret Cancer Centre (Toronto, Ontario, Canada). Written informed consent was obtained from all enrolled participants. The study was carried out in compliance with the principles of the Declaration of Helsinki and Good Clinical Practice standards. This post hoc correlative analysis, performed as part of an academic collaboration between Aqtual Inc. and the Princess Margaret Cancer Centre, also received approval from the institution’s Research Ethics Board (reference number: 18-6093.18).

### Endpoints

The primary objective was to assess (i) the feasibility of cfDNA_ac_ detection from baseline plasma and (ii) its association with clinical outcomes, including CBR and PFS by RECIST v1.1.

Secondary analyses characterized cfDNA_ac_-derived genomic features, including pathway enrichment, immune-related transcriptional signatures, CNV profiles, and tumor fraction, to identify potential predictive biomarkers of CPI benefit.

## Supplementary information

Supplementary Information

Supplementary Data

## Data Availability

The datasets generated and/or analyzed during the current study are not publicly available due to patient privacy considerations and institutional policies at the University Health Network (UHN) but are available from the corresponding author on reasonable request at albiruni.razak@uhn.ca. Access will be granted to qualified researchers for academic, non-commercial purposes in accordance with applicable ethical guidelines and institutional regulations.
