# Supplementary material for: Predicting immunotherapy benefit in leiomyosarcoma through active chromatin cfDNA profiling
Source: NPJ Precis Oncol. 2026 May 5;10:269. doi: 10.1038/s41698-026-01451-9 (PMC13350727; doi:10.1038/s41698-026-01451-9)
Supplement: Supplementary file 1 — Supplementary Information [file 41698_2026_1451_MOESM1_ESM.pdf]

Supplementary Information:

Supplementary Data 1. Clinical features and outcomes of patients with leiomyosarcoma included in the DAPPER trial

Median age (range)	54.5 (39-72) years old
Gender, n (%)<br>Female<br>Man	 28 (93.3)<br>2 (6.7)
LMS origin, n (%):<br>Uterine<br>Extra-uterine	 18 (60)<br>12 (40)
Median cycles of CPI-based treatment in the DAPPER trial (range)	3 (1-13)
Median PFS	2.8 (95% CI, 2.8-5.4)
Median OS	15.3 (95% CI, 14.7-15.4)
ORR, n (%)	2 (6.7)
CBR, n (%)	8 (26.7)
PD as best response, n (%)	22 (73.3)

Legend: PFS – progression-free survival; OS – overall survival; CBR – clinical benefit rate; PD – progression of disease.

Supplementary Data 2. Enrichment pathway analysis

Supplementary Data 3. Cancer-associated genes across differential CNV segments

Supplementary Data 4. Genes found in differential CNV segments

Supplementary Data 5. List of genomic features

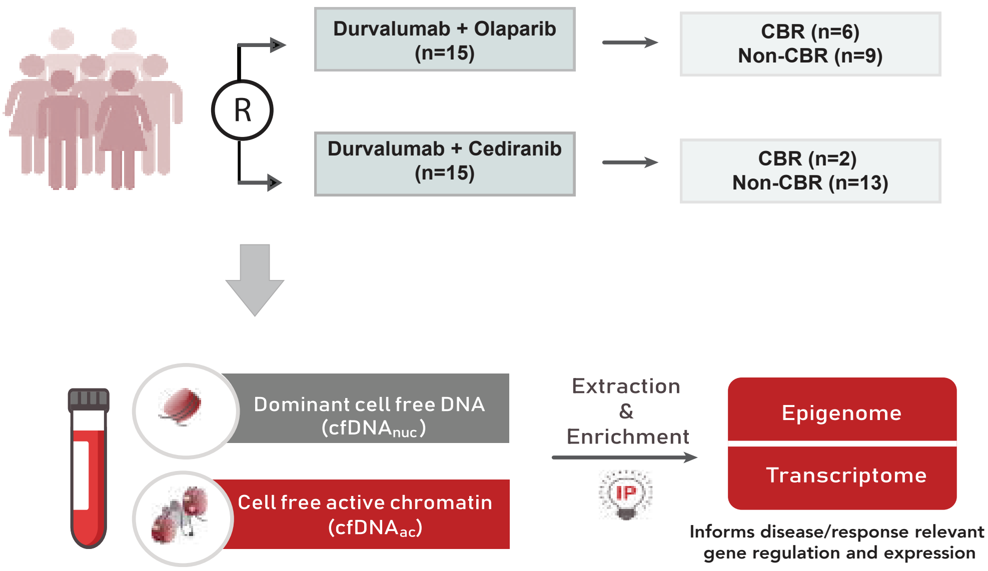

Supplementary Figure 1 | Consort diagram. Legend: CBR – clinical benefit rate.

A.

Features	Selected Features (n)
Promoter	305
Exon	615
Enhancer	95
Tissue TFBS	144
Immune TFBS	413
**Total**	**1570**

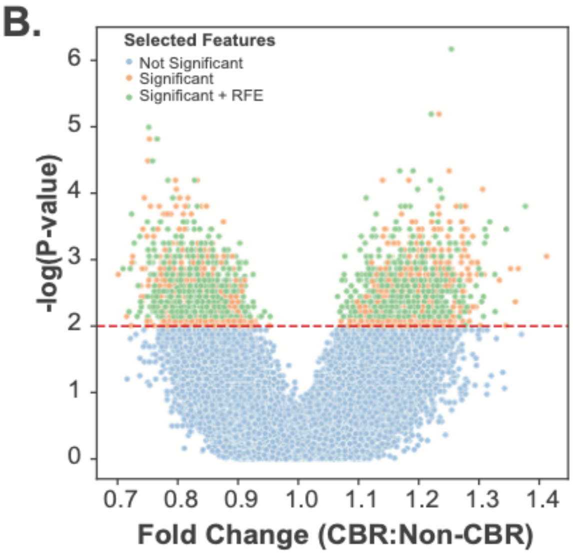

Supplementary Figure 2 | $cfDNA_{ac}$ feature selection workflow. (**A**) A total of 1,570 molecular features was selected across various genomic regions; **(B)** Feature selection was performed using the Mann-Whitney U test ($p < 0.01$) followed by recursive feature elimination (RFE) for refinement. Legend: CBR – clinical benefit rate. CBR and non-CBR groups had 8 and 22 patients, respectively.

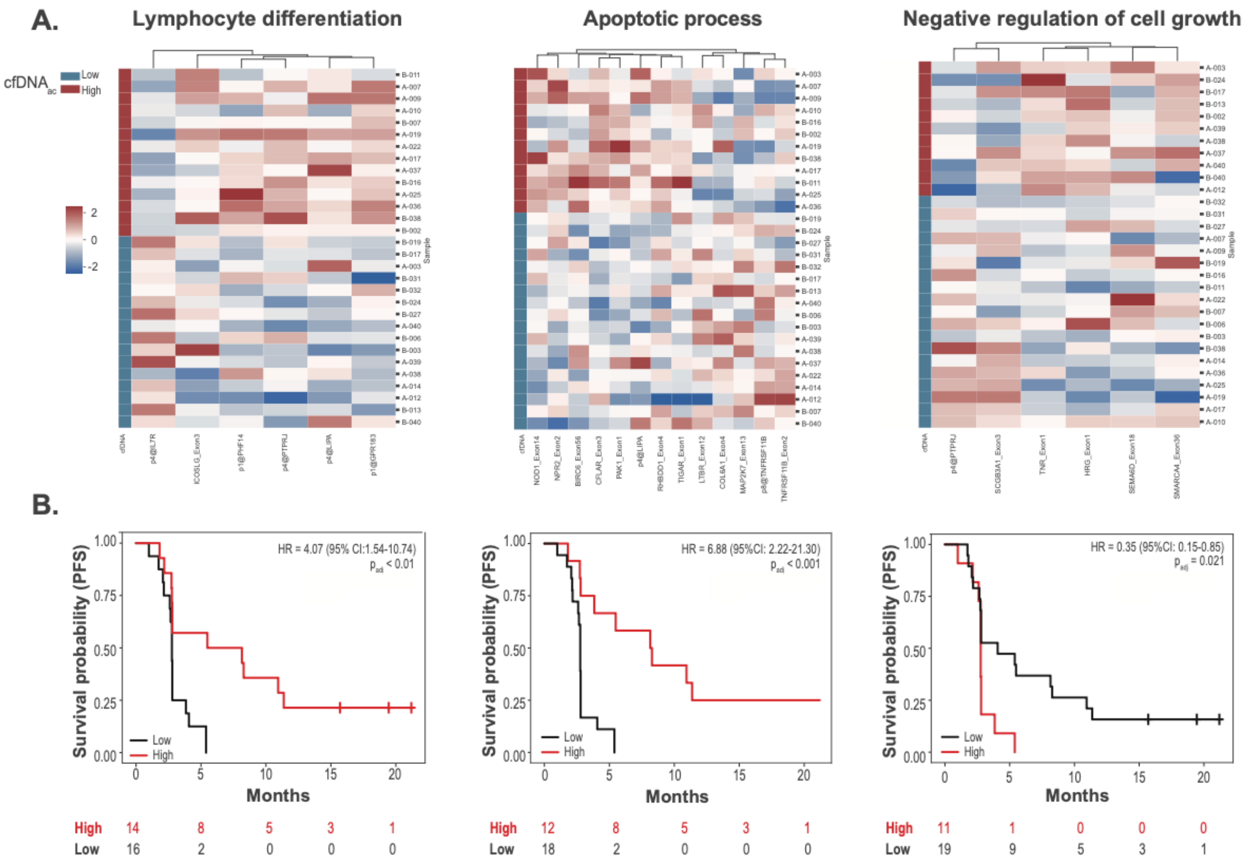

Supplementary Figure 3 | cfDNA$_{ac}$ signatures and clinical outcomes. (A) Heatmaps show the distribution of patient clinical outcomes stratified by lymphocyte differentiation, apoptotic process, and negative regulation of cell growth (top part, from left to right). All signatures are significantly associated with PFS after adjusting for FDR using the Benjamini–Hochberg method ($p < 0.05$). **(B)** Corresponding Kaplan–Meier curves illustrating PFS associations are shown below each heatmap. Legend: PFS – progression-free survival; FDR - false discovery rate.

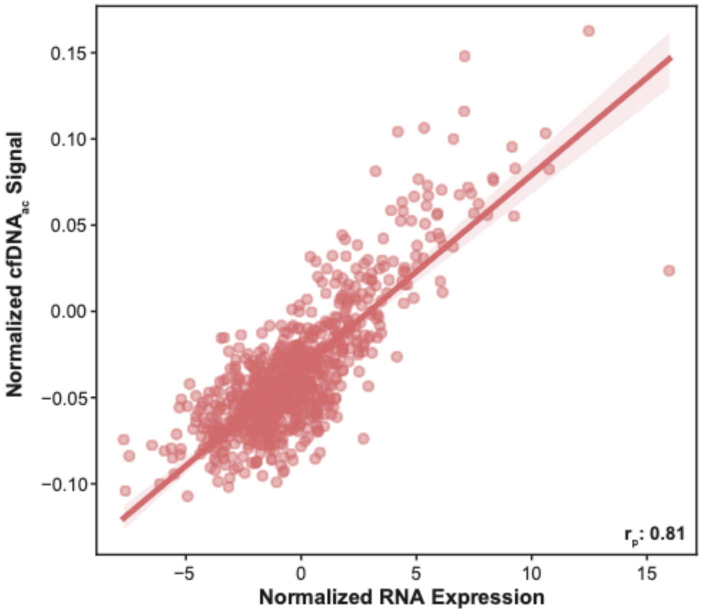

Supplementary Figure 4 | Correlation between tissue RNA expression and cfDNA$_{ac}$ signals. Comparison of tissue-derived RNA and active chromatin cfDNA signals was conducted using LMS baseline plasma samples (n=17) with matched tissue RNA-seq data. Tissue RNA expression levels and cfDNA$_{ac}$ signals were independently normalized to GTEx whole blood expression data and cfDNA$_{ac}$ profiles from healthy individuals, respectively. 7780 genes were considered in this analysis based on quality check of cfDNA and RNA-seq. Metagene profiles were generated using bins of size 10 for better noise reduction and trend detection.
